# Gastrointestinal Permeability After Bariatric Surgery: A Systematic Review

**DOI:** 10.7759/cureus.60480

**Published:** 2024-05-17

**Authors:** James W O'Brien, Nabeel Merali, Chris Pring, Tim Rockall, Denise Robertson, David Bartlett, Adam Frampton

**Affiliations:** 1 Department of Surgery, School of Biosciences and Medicine, University of Surrey, Guildford, GBR; 2 Department of Minimal Access Therapy Training Unit, Royal Surrey NHS Foundation Trust, Guildford, GBR; 3 Department of Bariatric Surgery, University Hospitals Sussex NHS Foundation Trust, Chichester, GBR; 4 Department of Nutrition, School of Biosciences and Medicine, University of Surrey, Guildford, GBR

**Keywords:** obesity and metabolic syndrome, intestinal dysbiosis, gastrointestinal microbiome, intestinal permeability, post-bariatric surgery, bariatric & metabolic surgery fbms

## Abstract

Gastrointestinal permeability refers to the movement of substances across the gut wall. This is mediated by endotoxemia (bacterial products entering the systemic circulation), and is associated with metabolic disease. The effect of bariatric surgery on permeability remains uncertain; the associated dietary, metabolic and weight changes are suggested to influence, or trigger, altered permeability. The primary aim of this study is to synthesize evidence and analyze the effect of bariatric surgery on permeability. A systematic review was performed, searching MEDLINE, EMBASE, and Scopus until February 2023, using MESH terms “intestinal permeability”, “bariatric”, for studies reporting in vivo assessment of permeability.

Three cohort studies and two case series were identified (n=96). Data was heterogeneous; methodology and controls preclude meta-analysis. Gastroduodenal permeability reduced post-sleeve gastrectomy (SG). Two studies showed an increase in small intestinal permeability after biliopancreatic diversion. Two studies revealed a decrease in post-Roux-en-Y gastric bypass. One study identified increased colonic permeability six months post-SG. Evidence regarding permeability change after bariatric surgery is conflicting, notably for the small intestine. Impaired colonic permeability post-SG raises concerns regarding colonic protein fermentation and harmful dietary sequelae. There are multiple interacting variables confounding gastrointestinal permeability change; procedure type, altered microbiota and metabolic response to surgery. Further understanding of this important aspect of obesity is required, both before and after bariatric surgery.

## Introduction and background

Bariatric surgery is established as an effective treatment for metabolic disease. It results in significant weight loss and ameliorates co-morbidities such as hypertension and type 2 diabetes mellitus (T2DM) [1]. Bariatric surgery reconfigures gastrointestinal anatomy and fundamentally modifies gut function; procedures are traditionally described as restrictive (sleeve gastrectomy (SG) and adjustable gastric band (AGB)) or malabsorptive (biliopancreatic diversion with or without duodenal switch (BPD/BPD-DS), and Roux-en-Y gastric bypass (RYGB)) [2]. The two most common procedures worldwide are RYGB and SG [3]. RYGB reduces stomach capacity to 20-40cm^3^ with diversion of gastric content to the distal small intestine [3]. SG creates a gastric tube by resection of up to 85% of the stomach, without modifying the small intestine [3]. Both procedures result in up to 30% total weight reduction [1,2]. Many studies describe the enteroendocrine hormone and anatomical changes, which are beyond the scope of this review. In brief, it is through an increase in glucagon-like peptide-1 (GLP-1), gastric inhibitory peptide (GIP) and peptide YY (PYY) that act to reduce food intake and confer satiety, with GLP-1 also interacting with central nervous system appetite reward pathways [3]. It is also clear that nutrient exposure, especially in the small intestine, is significantly altered following surgery, effectively reducing gut digestive function [4,5]. The gastrointestinal tract is the site of interaction between the immune system, glucometabolic pathways and the microbiome (bacterial, archaeal, viral, and eukaryotic microorganisms resident in the gut) [6-9]. It is therefore accepted that weight loss after surgery is multifactorial, involving many complex mechanisms in addition to neuro-enteroendocrine change [7].

Gastrointestinal permeability refers to the degree of movement of substances from the gut lumen across the epithelial wall, into the portosystemic circulation. This is mediated via endothelial tight junctions and passive diffusion through cells. Gut barrier function evolves in the first few months of life, and is necessary for normal digestion, immune function and cellular signaling [10-12]. An increase in permeability implies the passage of harmful macromolecules, such as microbiota-derived lipopolysaccharide (LPS), which is a glycolipid molecule in the cell wall of gram-negative bacteria [13,14]. A two- to threefold increase in serum LPS, in response to non-infectious stimuli, is described as endotoxaemia [15,16]. Studies suggest obesity and impaired barrier function are correlated, potentially via a lipid-rich, LPS-promoting obesogenic diet [17,18]. Acute critical illness, coeliac disease, inflammatory bowel disease (IBD), T2DM, metabolic-associated fatty liver disease (MAFLD) and cirrhosis have also been associated with endotoxaemia, dysbiosis (adverse microbiome change) and increased gastrointestinal permeability [19-25].

The effect of bariatric surgery on gastrointestinal permeability remains unclear. Koutoukidis et al. [26] calculate that per kilogram of weight loss via surgery or diet, there is a small standardized mean improvement in permeability, and surgical cohorts have shown conflicting changes in gastroduodenal, small intestinal and colonic permeability [27,28]. If weight loss following bariatric surgery occurs independently of improvement in gastrointestinal barrier function, endotoxemia may persist [14,29,30]. Patients may be at continued risk from the metabolic milieu associated with dysbiosis and impaired barrier function [31]. This has clinical importance when considering weight regain, post-operative metabolic and gastrointestinal health, and adds to uncertainty regarding the mechanisms of obesity [32-35]. This article aims to perform a systematic review of the evidence regarding gastrointestinal permeability change after bariatric surgery.

## Review

Methods

The Preferred Reporting Items for Systematic Reviews and Meta-Analysis (PRISMA) checklist was used to report this systematic review, with a pre-specified protocol [36]. The review was registered with PROSPERO (CRD42022314730).

Selection Criteria

Articles were screened against pre-determined inclusion criteria: (1) population: adults (>16 years old); (2) intervention: bariatric surgical intervention or procedure; (3) outcome: intestinal permeability measured using any in vivo method (as a primary or secondary outcome, assessed both before and after surgery); and (4) study design: any observational or interventional study, randomized or non-randomized. (5) if a comparator included: a lean control group or obese control group not undergoing bariatric intervention or procedure. The primary outcome was participants with any change to intestinal permeability following bariatric surgery. Exclusion criteria included (1) animal studies. There was no exclusion based on the quality of data, language, or publication type.

Eligibility Criteria and Search Strategy

Using the PICOS (Population, Intervention, Comparison, Outcome and Search Strategy) framework, the question was formed: “What is the effect of bariatric surgery on gastrointestinal permeability in humans”. P: adult humans. I: bariatric surgery of any type. C: control group; baseline or to other groups (e.g. lean BMI or non-operated participants). O: in vivo gastrointestinal permeability measured by any technique. S: any study design (due to the anticipated small number of results).

EMBASE, Scopus and MEDLINE were searched from inception until 1st February 2024. Search terms included BOLEAN operators and MESH terms encompassing gastrointestinal permeability, and bariatric procedures. The search in MEDLINE was adapted for other databases (see appendices). Prospero, Cochrane Library, ClinicalTrial.gov, International clinical trials platform (World Health Organization) and International Standard Randomised Controlled Trial Number (ISTCRN) registry were also searched to identify relevant protocols for trials and systematic reviews in this area. Grey literature (including conference abstracts) was searched, and all reference lists were checked, given the small number of studies. The search included articles in the English language only.

Data Analysis

Two independent reviewers (JO and NM) screened titles, abstracts, and full texts. No discrepancies were encountered. Quality of cohort studies was assessed using the Newcastle-Ottawa Quality Assessment Scale, and a modified version for case series [37,38]. No randomized or non-randomized trials were found. Data was collected for the year of publication, study design, number of participants and controls, body mass index (BMI), bariatric intervention, follow-up, permeability assessment method and results. Data was recorded using Microsoft Excel 2017 (Microsoft, Redmond, WA, USA). Data extraction tables were used to summarize the key findings of all eligible studies. Significant heterogeneity in study design and permeability assessment means quantitative comparison and data pooling are unreliable and at risk of significant bias. A meta-analysis could not be performed.

Results

Study Selection

**Figure 1:**
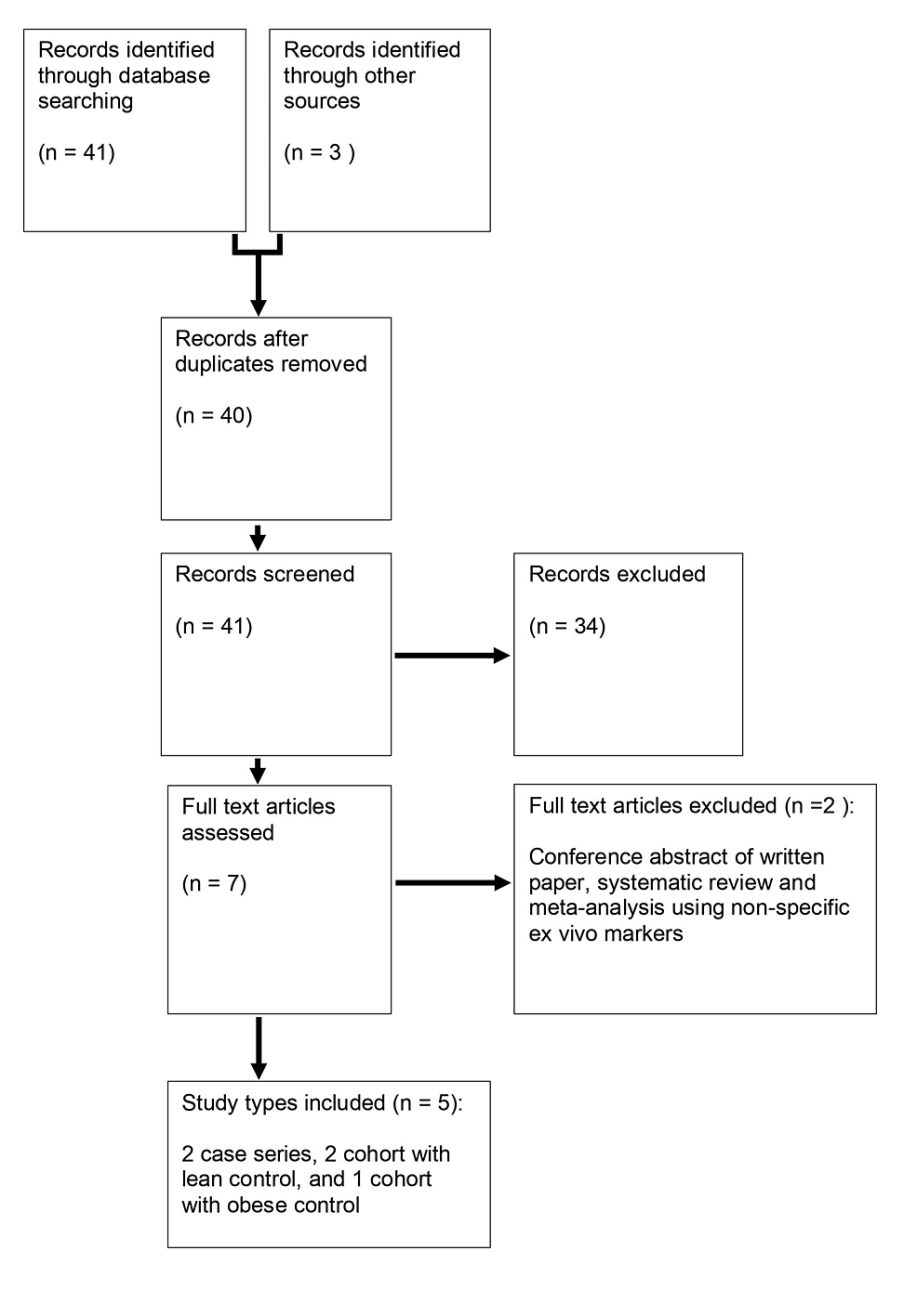
PRISMA flowchart PRISMA: Preferred Reporting Items for Systematic Reviews and Meta-Analysis Reference [36]

The PRISMA flowchart (Figure 1) summarizes the outcome of the search strategy. From screened titles, two case series and three cohort studies (with control groups) were included. Using the Newcastle-Ottawa Quality Assessment Scale, the studies scored poorly for comparability of cases and controls, and causality (Tables 1-2).

**Table 1:** Newcastle-Ottawa scale for cohort studies (Newcastle-Ottawa Scale, reference [37])

Study	Selection (4)	Comparability (2)	Outcome (3)
Kellerer [27]	****	**	***
Carswell [28]	****	*	***
Wilbrink [39]	***	**	***

**Table 2:** Adapted Newcastle-Ottawa scale for case series Q1: Does the patient(s) represent(s) the whole experience of the investigator? Q2: Was the exposure adequately ascertained? Q3: Was the outcome adequately ascertained? Q4: Were other alternative causes that may explain the observation ruled out? Q7: Was follow-up long enough for outcomes to occur? Q8: Is the case(s) described with sufficient details to allow other investigators to replicate the research or to allow practitioners make inferences? (Questions 5 and 6 are relevant to cases of adverse drug events and were excluded) Reference [38]

Study	Selection (/1)	Ascertainment (/2)	Causality (/2)	Reporting (/1)	Total (/6)
Savassi-Rocha [40]	1	2	1	1	5
Gaggiotti [41]	1	2	1	1	5

Study Characteristics

The study characteristics and methods are summarized in Table 3. All are from European institutions, with four studies published in the last decade. Four studies assessed baseline permeability (pre-operative cohort), and follow-up ranged from early (one week to three months) to late (six months) [27,28,39,40]. Carswell et al. followed up at 20 months [28]. Three studies used a control group [27,28,39]. All authors except Gaggiotti et al. [41] stated the operative approach. In total, 96 participants were analyzed pre-operatively. Participants underwent laparoscopic SG (44), RYGB (16 open, seven laparoscopic), BPD (18), AGB (six) and laparoscopic BPD-DS (five). In total, 74 participants were followed up after surgery. There were 51 controls at baseline (seven obese and 44 lean). All studies reported significantly lower BMI at follow-up. The mean BMI of the operated participants was 50.3kg/m^2^ at baseline, with a mean reduction after surgery of 13.42kg/m^2^.

**Table 3:** Study demographics and methods AGB: adjustable gastric band; RYGB: Roux-en-Y gastric bypass;* *BPD(-DS): biliopancreatic diversion (with duodenal switch); SG: sleeve gastrectomy; GD: gastroduodenal; SI: small intestine; C: colon; BMI: body mass index (kg/m^2^) *BMI (kg where stated), **nine operated followed up, ***14 operated followed up

First author (year)	Study design	Number of participants operated and controls	Surgery	Longest follow-up (months)	Weight at baseline (follow-up)*	Segment tested: sugar probe (collection period (hours))
							Kellerer (2019) [27]	Cohort (lean control)	17 operated	SG	6	52.5 (39.1)	GD: sucrose
17 controls		21.5	SI: lactulose/mannitol (0-5)								
			C: sucralose (5-21)								
Carswell (2014) [28]	Cohort (obese control)	18 operated	6 AGB	20	44 (38)	SI: lactulose/L-rhamnose, D-xylose (0-5)
			7 RYGB		47 (36)	
			5 BPD-DS		60 (30)	
		7 controls			47 (47)	
Wilbrink (2019) [39]	Cohort (lean control)	27 operated***	SG	6	45.4 (38.7)	GD: sucrose
		27 controls			22.9	SI: lactulose/L-rhamnose (0-5)
						C: sucralose/erythritol (5-24)
Savassi-Rocha (2013) [40]	Case series	16 operated	RYGB	6	52.9 (-26.6kg)	SI: lactulose/mannitol (0-5)
Gaggiotti (1995) [41]	Case series	18 operated**	BPD	6	47 (-31.8kg)	SI: lactulose/mannitol (0-5)

Assessment of Permeability

Results were categorized into three discrete functional sections of the gastrointestinal tract: gastroduodenal, small intestinal and colonic (Table 4). By examining the urinary excretion of oral sugar probes using high-performance liquid chromatography, intestinal permeability was expressed per sugar, as a percentage of the ingested amount. Sugars are established for estimating permeability in vivo and are specific for each section of the gastrointestinal tract [42]. Four studies reported median and range [27,28,39,40], and Gaggiotti et al. quoted mean [41]. It is suggested that each laboratory use healthy controls to determine the normal range [43]; therefore all studies compared results within a cohort, or to controls. Of the included studies, two assessed gastroduodenal permeability using sucrose [27,39] and the same two studies assessed colonic permeability using sucralose and sucralose/erythritol. To assess small intestinal permeability all five studies used lactulose; three combined it with mannitol [27,40,41], and two with rhamnose [28,39], to generate a lactulose/mannitol or lactulose/rhamnose ratio. All the studies used repeated measure analysis of variance (ANOVA) to compare between groups, and a variety of other tests to assess correlations, such as the Mann-Whitney test for paired data. Each study described a pre-defined level of statistical significance associated with each test.

**Table 4:** Results of permeability testing *mean values (%), **ratio, empty cells: N/A T0: baseline; AGB: adjustable gastric band; RYGB: roux-en-y gastric bypass; BPD(-DS): biliopancreatic diversion (with duodenal switch); SG: sleeve gastrectomy; GD: gastroduodenal; SI: small intestine; C: colon

First author (year and cohorts)		Results							Significant findings (permeability)					
		GD*		SI**			C**		GD		SI		C	
		T0	Follow-up	T0		Follow-up	T0	Follow-up	T0	Follow-up	T0	Follow-up	T0	Follow-up
Kellerer (2019) [27]	SG	0.18	0.08	0.03		0.02	0.40	1.24		Significantly reduced (p=0.012)				Significantly increased (p=0.002)
	Controls	0.20		0.02			0.56		Significantly increased (p=0.003)				Significantly reduced (p=0.006)	
Carswell (2014) [28]	BPD-DS					0.117						Significantly increased (p<0.02)		
	AGB					0.003								
	RYGB					0.010								
	Controls					0.014								
Wilbrink (2019) [39]	SG	0.51	0.23	0.029	0.047		0.049	0.04	Significantly increased (p<0.05)			Significantly increased (p<0.05)		
	Controls	0.20												
Savassi-Rocha (2013) [40]	RYGB			0.0136		0.0172								
Gaggiotti (1995) [41]	BPD			0.019	0.022							Significantly increased (p<0.05)		

Outcomes of Permeability Testing

Gastroduodenal: Kellerer et al. describe a significant reduction in gastroduodenal permeability post-SG compared to baseline (0.08% from 0.18% (p=0.012)); at baseline, there was no difference to lean controls (0.18% vs 0.20% (p=0.95)) [27]. Wilbrink et al. also report a reduction post-SG [39]. At baseline gastroduodenal, permeability was significantly increased in the 27 operated participants compared to lean controls (0.51% vs. 0.20% (p<0.05)). In the 14 participants followed up after surgery, permeability reduced to become no different to lean controls (at baseline), but the reduction within the operated cohort was not significant (0.3% from 0.49% (p=0.09)) [39]. It can be tentatively suggested that following SG, there is a reduction in gastroduodenal permeability from baseline. The similarity in method and cohort selection between the two studies means this conclusion is less susceptible to bias. No conclusion can be drawn from the other segmental analyses, due to the risk of bias relating to study design.

Colonic: The same authors also assessed colonic permeability after SG. Kellerer et al., using sucralose, noted a significant increase in colonic permeability at six months follow-up (1.24% from 0.40% (p=0.002)); at baseline, there was no difference to lean controls [27]. Wilbrink et al., using a sucralose/erythritol ratio, found no overall difference in follow-up within the operated group (0.04 from 0.049), or at baseline compared to lean controls [39]. A single colonic probe (erythritol) significantly reduced in follow-up (23.4% from 28.6% (p<0.05)); the clinical significance of this is unclear.

Small intestinal: All studies assessed the small intestine (reporting individual sugars and the ratio of lactulose with mannitol or L-rhamnose). Two describe an increase in permeability following malabsorptive procedures [28,41]. Gaggiotti et al. report small intestinal permeability significantly increased one week after BPD compared to baseline (0.18 from 0.01 (p<0.05)), but this returned to baseline in the participants followed up at six months [41]. Carswell et al. report increased permeability following laparoscopic BPD-DS (0.117) when compared to AGB (0.003), RYGB (0.01) and obese controls (0.014) at 20 months (all p<0.02); there was no comparison to baseline [28]. Wilbrink et al. describe increased small intestinal permeability at six months post-SG (0.047 from 0.029 (p<0.05)); there was no significant difference to lean controls at baseline or in follow-up [39].

Two studies describe reduced small intestinal permeability. Savassi-Rocha et al. report decreased permeability one-month post-RYGB compared to baseline, for a single sugar (mannitol) (6.73% from 10.89% (p=0.003)), which became no different to baseline at six months [40]. Kellerer et al. report decreased permeability at six months post-SG, compared to baseline, again, only for mannitol (8.70% from 12.43% (p=0.012)) [27]. There was not a statistically significant change in the lactulose/mannitol ratio in either study.

Discussions

There is conflicting evidence regarding permeability change in patients undergoing bariatric surgery. It is evident that a strong degree of heterogeneity exists between study cohorts, methodology and follow-up. Further research is required before definitive conclusions can be drawn. Small intestinal permeability was analyzed in all four studies. The patients undergoing more malabsorptive surgery (BPD and BPD-DS) showed an increase (deterioration) in small intestinal permeability. There was no consensus for patients undergoing SG and RYGB. Both papers assessing gastroduodenal permeability showed a reduction (improvement) in permeability. Colonic permeability was assessed in two cohorts undergoing SG [27,39]; Kellerer et al. reported a deterioration in colonic barrier function after surgery, despite significant weight loss. This is contrary to the finding by Di Palo et al. [44], that deteriorating colonic permeability correlated with increasing BMI in a non-operated cohort.

This colonic finding may have clinical implications; post-operative advice after bariatric surgery can include proportionally higher protein intake [45]. When combined with altered gut physiology [46], this may expose the colon to ≥17g/day of protein/day [47]. Colonic protein fermentation is known to generate proportionally more potentially carcinogenic ammonia and sulfur-containing compounds, indoles, and phenols [48-50] and proportionally less of the immuno-protective short-chain fatty acid, butyrate [49]. These substances have been associated with compromised epithelial integrity, loss of the mucus layer and increased colonic permeability in vitro [51-53]. Despite increasing acceptance that overall colonic cancer risk reduces post-surgery, the evidence remains conflicting for rectal cancer [33-35]; further investigation of this colonic finding is warranted.

Over the last 20 years, in vivo analysis of permeability using sugar probes has become established as the easiest and most reliable method in the clinical setting [42,54-56]. Administered orally, the probes are completely excreted, either passing through the entire gastrointestinal tract unchanged in feces, or by moving across the bowel wall into the systemic circulation. Aside from sucralose, which is found in sweeteners, negligible amounts of each probe are found in the normal diet. With no further hepatic or renal metabolism, the sugars are excreted unaltered in urine. By assessing the quantity of sugar in urine (sampled at time intervals following oral administration), the probes can act as a marker of gut wall permeability. Increased urinary concentration represents increased permeability. Similar functional tests utilizing a radioisotope probe chromium‐51 ethylenediamine tetraacetic acid (^51^Cr-EDTA) are reliable, but expensive [57,58].

Two transport mechanisms control gastrointestinal permeability. Paracellular transport consists of movement through intercellular junctions, or across endothelial tight junctions (protein complexes) [59]. In oral sugar probe testing, this is demonstrated by the large disaccharide lactulose. Transcellular transport is passive diffusion through cells, and is represented by the small monosaccharides mannitol or L-rhamnose [42]. Lactulose, mannitol and L-rhamnose undergo colonic fermentation, making them specific to the small intestine [42]. A ratio of two probes is used to mitigate pre-mucosal confounding factors such as gastric emptying, dilution, intestinal transit, and bacterial overgrowth. Post-mucosal confounding factors include co-morbidity such as renal disease. A ratio generates an index of small intestinal permeability to correct for an (assumed) equal effect of the confounding factors [22]. Kellerer and Wilbrink et al. used sucrose, which rapidly degrades in the small intestine and is specific for gastroduodenal permeability [27,39,54].

The same authors investigated the colon using sucralose and the ratio of sucralose/erythritol. These sugars undergo minimal absorption and degradation throughout the gastrointestinal tract and are colon-specific [42,54-56,60]. Kellerer et al. analyzed plasma zonulin, a tight junction modulator protein, previously widely studied as a marker for intestinal permeability [27]. Concerns persist regarding the sensitivity and applicability of this commercially available enzyme-linked immunoassay (ELISA) and therefore it was not considered in this analysis [61,62].

It is largely accepted that urine analysis three to six hours after oral ingestion reflects small intestinal permeability, and eight or more hours represents colonic permeability [42]. There is evidence that these time points may be unreliable; some authors suggest that lactulose/mannitol requires interpretation at 2.5-4 hours [63], or even that excretion ratios should be discarded [55]. As urinary mannitol can be detected over a long period, they recommend reporting a single absolute sugar value at 0-2 hours for the small intestine and 8-24 hours for the colon [55,64]. In this review, all the studies assessed small intestinal permeability for up to five hours. Two studies included colonic permeability at 21 and 24 hours [27,39].

Interpretation of the small intestinal sugar probes varied between studies, with three reporting altered lactulose/mannitol ratio [27,40,41]. Two studies described reduced permeability (six months post-SG and one month post-RYGB) from single sugar values (the ratio was unchanged) [27,40]. Inferences were made based on the single sugar probe. Little consensus exists regarding the interpretation of individual sugar probes without a change in ratio. It has been suggested that a ratio is required to conclude clinically significant changes in permeability [42].

All the studies expressed reservations regarding the impact of surgery on pre-mucosal factors. Altered gastrointestinal transit post-bariatric surgery is a significant confounding factor [65]. The heterogeneity of bariatric procedures complicates analysis and is difficult to mitigate, as studies demonstrate increased gastric emptying and intestinal motility after SG and no change following RYGB/DS [2,5,65-69]. Carswell et al. used sulphapyridine, a marker of oro-caecal transit time, with no change demonstrated [28]. A second significant confounder is the non-standardized size of the gastric tube in SG, and variation in alimentary/biliary limb length in RYGB/DS [70-73].

Causes of Altered Permeability

The microbiome significantly contributes to gut barrier function [74,75], through maintenance of the mucus layer (mechanical barrier) and by processing dietary intake to provide an energy source for epithelial cells [75-80]. It is postulated that disturbed gastrointestinal permeability results from a combination of, or interaction between, genetic defects in the immune and barrier function of the gut epithelium, and environmental risk factors such as diet and infection [81].

Studies have attempted to clarify the role of harmful bacterial products, particularly LPS, in the context of impaired permeability. LPS moves via passive diffusion from the gut lumen into epithelium, binding with chylomicrons or lipopolysaccharide-binding protein (LPB) for transport to the liver for clearance. During this, Toll-like receptor four (TLR4) binds LPS, particularly in lymph nodes [82]. In short, a potent host immune response is propagated, ultimately activating signalling pathways that are associated with inflammatory disease, diabetes, and obesity, such as JNK1 and NF-kB [15,83-86]. Kellerer et al. found plasma LPB was increased at baseline but did not reduce post-SG [27]. This is in keeping with other human studies, which have not reproduced the exaggerated LPS response demonstrated in critical illness or animal models of endotoxaemia [13]. This is possibly because the increase in LPS of obesity is thought to be more subtle (by a factor of 10-50) [85].

The microbiome data in this review is limited. Savassi-Rocha et al. excluded participants with bacterial overgrowth, acknowledging that treatment of small intestine bacterial overgrowth has been associated with improvement in permeability [40,87]. A single study (Kellerer et al.) [27] examined the microbiome via 16S rRNA gene sequencing, describing lower alpha-diversity in the obese cohort at baseline, with a wide variety of findings across several other species (alpha-diversity increased in some participants post-SG). Animal and human studies demonstrate lower overall diversity in fat-rich diet and obesity, with gram-negative Bacteroidota reduced and gram-positive Firmicutes proportionally increased, and Bacteriodota recovering after weight loss (through low-calorie diet or bariatric surgery) [26,88-92].

Limitations

As this review included only studies reporting in vivo assessment of permeability, combining the sugar ratios and absolute values generated by mass spectrometry of urine could permit meta-analysis. There was minimal heterogeneity in methodology and statistical analyses, as each study observed the established protocol for the use of sugar probes [55]. However, due to the high level of heterogeneity in patient selection, pooling of data will result in a significant risk of bias. This review yielded a small number of studies, containing three different cohorts, six bariatric interventions and seven different follow-up intervals. Kellerer et al. [27] and Wilbrink et al. [39] were the only two investigating the same procedure (SG). Each study analyzed the operated cohort in various ways. Baseline, follow-up, lean and obese control groups, and different procedures were compared. There is a significant difference in anatomical change between procedure types [2]. Given this lack of standardized study methodology, there is a risk of bias when grouping the statistical findings, which can only be compared for the overall direction of permeability change. Comparing an operated cohort to baseline (after significant weight loss) is not comparable to a lean or obese control group not undergoing surgery; there is evidence that alteration in permeability can occur independently of weight [14,26]. In summary, due to the complexity of the field and limited existing evidence base in bariatric cohorts, the included studies were not able to control for the many factors that confound permeability assessment. Future cohorts should investigate a single procedure, similar to Kellerer and Wilbrink [27,39].

The studies assessed additional gastrointestinal and systemic markers; two reported faecal calprotectin and plasma citrulline [28,39]. Two studies measured faecal short-chain fatty acids and plasma non-esterified fatty acids [27,28]. Pooling of these secondary outcomes is not possible due to the paucity of data. A published meta-analysis of weight loss and permeability was excluded; the authors included seven studies that reported changes in plasma LPS and LPB after bariatric surgery [26]. Whilst associated with metabolic dysfunction and the endotoxaemic process, these markers are significantly associated with dietary fat and acute phase inflammation and so are not specific for gastrointestinal permeability [29].

Different mechanisms were suggested by the authors, reflecting the heterogeneous design of the included studies. Two authors [40,41] suggest initial improvements in intestinal permeability were not sustained because of intestinal adaption (minimizing malabsorption post-surgery). The functioning of epithelial tight junctions in the context of epithelial hyperplasia is complex, and frequently cited evidence from IBD cohorts may not translate to obesity [93]. Wilbrink et al. [39] suggest that proximal gut inflammation post-procedure impaired gastroduodenal permeability. Barrier integrity has been correlated with the focal site of inflammation in IBD and coeliac disease previously [23], but the increase in faecal calprotectin in the operated cohort was within the normal range. Two studies discussed accelerated gastric emptying and small bowel transit [27,39]. The effect of SG on gut physiology is unclear [67,68], and is adjusted for by reporting ratios, rather than individual sugars. Savassi-Rocha et al. [40] highlight that permeability may not be directly dependent on weight or surgery, but rather change to the pre-operative high-fat diet, caloric restriction, consecutive weight loss and the microbiota. These factors are known to contribute to the endotoxaemic process [13,85].

All studies reported at least six months of follow-up. Carswell et al. [28] included a wide range of time periods for each procedure (AGB, RYGB and BPD-DS), confounding comparison, and excluded one participant due to a diagnosis of ulcerative colitis. Gaggiotti et al. [41] followed half of the cohort (9/18). Six-month follow-up may not adequately represent sustained permeability change; it has been demonstrated that small intestinal permeability continued to decrease between six and 12 months after initial weight loss (even if weight remained stable or increased) [94]. Two studies outlined a power calculation [27,39], based on permeability assessment in non-bariatric cohorts [92].

Future Applications

The rate of weight recidivism (initially >50% excess weight loss (EWL) but regain to <50% EWL) is reported to be as high as 27.8% after surgery [95], and revisional surgery comprises 10.5% of all bariatric procedures worldwide [96]. Post-operative treatment is emerging, such as the use of post-operative GLP-1 receptor agonists; Mok et al. report 8.03% further body weight reduction in those responding poorly to surgery [97]. Therefore, optimization prior to surgery warrants increasing focus. This is a significant responsibility due to the increasing volume of bariatric surgery performed worldwide (311, 441 procedures in 2020/21) [98]. The rapidly evolving field of “precision nutrition” argues that individual factors such as endotoxaemia and gut health are the main modifiable determinants of metabolic health [96]. Individualized assessment of many endotoxaemic markers, for example, dietary health and microbiome, is commercially available, but collection of urine for in vivo gastrointestinal permeability analysis remains impractical [95]. There is evidence that modifying the endotoxaemic process, for example, treatment with antibiotics or probiotics, can improve metabolic dysfunction; reduce colonic LPS [99], induce remission and improve dysbiosis [100,101] and reduce disease activity in IBD [102]. Similar treatment post-bariatric surgery may mitigate the potential for the injurious colonic endotoxaemia identified by two of the studies in this review [27,39].

## Conclusions

The overall direction of permeability change after bariatric surgery remains uncertain, particularly with regard to the small intestine. Altered permeability following bariatric intervention appears dependent on multiple interacting variables, including the microbiome, anatomical reconfiguration, and changing gut metabolic function. The conflicting findings highlighted demonstrate the difficulty in controlling for confounding factors and the multi-faceted gut-metabolic mechanism that underpins permeability. A standardized methodology is needed to allow meaningful comparison between studies. For each procedure, multi-sugar testing at six months follow-up, combined with 16S rRNA gene sequencing for microbiome analysis, will advance understanding. Investigation into the impaired colonic permeability post-SG reported by a single study is warranted, considering underlying carcinogenic potential and the clinical implication of increased dietary protein intake after surgery. Further study of gastrointestinal permeability change, endotoxaemia and surgery, will ultimately allow a greater appreciation of the intertwining mechanisms of metabolic disease, obesity, and gastrointestinal health.

## Appendices

Search strategy in EMBASE

1.    exp *"BARIATRIC SURGERY"/

2.    ("gastric sleeve" OR "sleeve gastrectomy" OR "gastric bypass" OR biliopancreatic OR (gastric ADJ band*) OR "roux-en-Y" OR "roux en Y" OR bariatric* OR ileojejunal).ti

3.    (1 OR 2)

4.    *"INTESTINE MUCOSA PERMEABILITY"/

5.    ((gut* OR gastrointestin* OR gastro-intestin* OR colon* OR intestin* OR jejun*) ADJ1 permeab*).ti

6.    (4 OR 5)

7.    (3 AND 6)

Search strategy in MEDLINE and SCOPUS

(((permeab* ADJ1 (intestin* OR colon* OR bowel* OR duoden* OR jejun* OR ileum OR ileal)).ti,ab OR (absorb* ADJ2 (intestin* OR colon* OR bowel* OR duoden* OR jejun* OR ileum OR ileal)).ti,ab OR (absorption ADJ2 (intestin* OR colon* OR bowel* OR duoden* OR jejun* OR ileum OR ileal)).ti,ab OR (exp *PERMEABILITY/ AND exp *"INTESTINAL MUCOSA"/)) AND ((bariatric* OR obes* OR diabet* OR overweight OR over-weight OR "high body-mass index" OR "high body mass index" OR "high BMI" OR "body mass index over" OR "body-mass index over" OR "bmi over" OR "metabolic syndrome").ti OR (exp *"DIABETES MELLITUS, TYPE 1"/ OR exp *"DIABETES MELLITUS, TYPE 2"/) OR exp *OVERWEIGHT/ OR exp *OBESITY/ OR exp *BARIATRICS/ OR exp *"BARIATRIC SURGERY"/ OR exp *"METABOLIC SYNDROME"/))
